# CyanoStat: An open-source platform for CO_2_ regulation in microbial incubators optimized for cyanobacterial cultivation

**DOI:** 10.1016/j.ohx.2025.e00649

**Published:** 2025-04-12

**Authors:** Tijn O. Delzenne, Dennis Claessen

**Affiliations:** Institute of Biology Leiden, Leiden University, Sylviusweg 72, 2333 BE Leiden, the Netherlands

**Keywords:** Carbon dioxide, Cyanobacteria, Microbial incubation, Controller

## Abstract

Accurate and reliable control of CO_2_ is essential for the incubation of many microbial organisms. Photosynthetic microbial organisms, such as cyanobacteria, pose a particular challenge, requiring both light and elevated CO_2_ concentrations for efficient growth. These characteristics highly limit the availability of affordable and reliable incubation devices, deterring laboratories from undergoing proof-of-concept research into cyanobacteria. To combat this, this study presents CyanoStat: an affordable add-on device for off-the-shelf microbial incubators. By consistently measuring the CO_2_ concentrations in an incubator, control is achieved by opening and closing the flow of gas from a pressurized bottle to the incubator. Through a tuned time-based valve control system, the device is able to rival the accuracy and stability of significantly more expensive hardware. Multiple implemented safety features allow for reliable usage over prolonged periods. CyanoStat, as described in this work, was able to achieve precise CO_2_ control with fluctuations of 120 ppm, 145 ppm and 260 ppm around a setpoint of 1, 2 and 5%, respectively. This demonstrates that the system is well-suited for both short-term proof-of-concept studies and long-term cultivation applications.

## Graphical abstract

**Figure:**
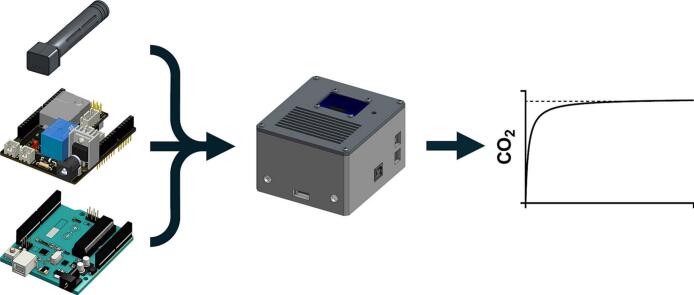

**Specifications table**

**Table:** 

Hardware name	CyanoStat
Subject area	• Biological sciences (e.g., microbiology and biochemistry) • Educational tools and open-source alternatives to existing infrastructure
Hardware type	• Measuring physical properties and in-lab sensors • Biological sample handling and preparation
Closest commercial analog	No commercial analog available
Open-source license	Creative Commons Attribution 4.0 International (CC BY 4.0)
Cost of hardware	€65
Source file repository	https://doi.org/10.17605/OSF.IO/ANM7H

## 1 Hardware in context

Cyanobacteria can capture carbon dioxide from the air and synthesize sugars using light as an energy source, allowing them to grow without the need for additional sources of carbon. This makes them a more sustainable alternative to the currently used heterotrophic bacterial hosts, which rely on sugar-rich media for growth and compound production. Recent advances have provided a slew of sophisticated tools for genetically engineering a number of model cyanobacteria [[1], [2], [3], [4], [5], [6]]. This has opened possibilities for renewable biosynthesis of a variety of valuable compounds, including ethanol [7], fatty acids [8,9], sucrose [10], and bioplastics [11]. However, advancements in the efficiency and scale of cyanobacterial research are needed to mature cyanobacterial tools and fully realize their potential for large-scale, sustainable bioproduction.

A major obstacle preventing laboratories from switching from heterotrophic to phototrophic bacteria is the significant initial investment in the required specialized equipment. Phototropic bacteria necessitate incubators that can supply light and CO_2_, crucial for efficient growth using a photosynthetic metabolism [12,13]. Equipment with such capabilities often comes with a price tag several times higher than that of an incubator designed for heterotrophic growth. This financial barrier may discourage laboratories from pursuing initial proof-of-concept research lines. While the addition of photosynthetic light to an incubator can easily be realized by installing LED lights originally fabricated for plant growth, CO_2_ regulation isn’t as easy to implement. To address this obstacle, we determined the need for development of a cost-effective CO_2_ regulation platform capable of adapting existing heterotrophic incubation devices towards the use for phototropic growth.

While several systems for the control of CO_2_ concentrations have been made before, these are often intended to be used temporarily [[14], [15], [16]]. For example, some of these devices—employing dry ice as a CO_2_ source or repurposing egg incubation chamber for heat—are specifically designed to enable short-term incubation under controlled gas concentrations. However, for long-term cultivation, these setups will prove to be rudimentary, lacking the reliability and low-maintenance functioning of a purpose-made microbial incubator. To overcome this obstacle, we developed a reliable and cost-effective CO_2_ controller, called CyanoStat, which can be added to most standard laboratory incubators, thereby facilitating the cultivation of CO_2_-dependent organisms. The system is designed as a stable, reliable, and easily adaptable platform for microbial incubation under elevated CO_2_ conditions, with parts costing less than €70. Thanks to its low cost, CyanoStat can easily be assembled, making it ideal for small-scale proof-of-concept studies with limited budgets or for cost-effectively expanding existing infrastructure. Although the system was optimized for cyanobacteria, it can also be applied to cultivate various eukaryotic organisms requiring elevated CO_2_ levels, such as microalgae and mammalian cell lines.

To allow for the simple implementation of CyanoStat, we offer C++ code for Arduino (compatible) microcontrollers, PCB designs, and CAD models for fabrication of purpose-made enclosures, along with an extensive assembly and setup guide. This allows for the replication of the device with minimal electronics and coding expertise required. Furthermore, we provide alternative methods for obtaining potential hard-to-get components and consumables required for a fully functioning system, with the goal of making the device more accessible to labs with a less developed infrastructure.

## 2 Hardware description

### 2.1 Design considerations

#### 2.1.1 Bang-bang control

Bang-bang control is a simple feedback control system that makes use of the switching between two extreme values based on the difference between a measurement and a setpoint value, also known as the error. When the measured value is perceived to be below a pre-determined setpoint value, the system will apply one extreme value. When the desired value is subsequently reached, the system will return the opposite extreme value. Because of the use of these extreme values, being either fully on or fully off, the variable regulated using this control method usually oscillates around the setpoint. The magnitude of these oscillations is determined by the dynamics of the system controlled, namely delays between an undertaken action and the subsequent measurement of the changes caused by this action. Although this control methodology's simplicity allows its implementation to be cheap, it inherently lacks the capability to achieve a fully stable state at the desired setpoint.

#### 2.1.2 PID control

PID or Proportional-Integral-Derivative control provides a more sophisticated mode of regulation than a bang-bang controlled system. This algorithm does not only consider the current deviation of a measurement from a setpoint, but also the length of time it has been since the setpoint was last reached and the rate of change towards or from the setpoint. The output of the proportional term is, as the name implies, directly proportional to the deviation from the desire setpoint, increasing as the error increases. The integral term sums up the error over time, accumulating to generate a corrective action that keeps increasing as long as the error persists. Lastly, the derivative term predicts future errors by taking into account the rate of change of the error signal. This allows the system to counteract rapid changes between the measurement and setpoint while minimizing oscillations and overshoot. The control signal from a PID controller is obtained by taking the sum of each of these signals multiplied by their respective gain settings. Tuning the gain of each of these terms determine how aggressively they impact the control signal and is required to reach optimal control over a system. The PID control signal is calculated using the following equation:

$$u\left(t\right)=K_{p}e\left(t\right)+K_{i}\;\int e\left(t\right)dt+K_{d}\frac{\mathrm{de}(t)}{\mathrm{dt}}\;$$

Here, *u(t)* is the control signal at time *t*, *e(t)* is the error signal at time *t* (calculated using setpoint − measurement), and *K_p_, K_i_* and *K_d_* are the proportional, integral, and derivative gains, respectively. While this system can lead to greater accuracy and stability, it requires more expensive components and much greater complexity than a bang-bang controlled system. The need for tuning of the gain parameters requires users to do experimental testing to reach stable regulation of the response variable, lowering the user-friendliness of the product implementing a PID control scheme.

### 2.2 System design

CyanoStat is designed for continuous use over prolonged periods of time, supporting both proof-of-concept research as well as functioning as a cheap expansion to increase the cyanobacterial incubation capacity of a growing laboratory. The system implements an off-the-shelf Arduino (compatible) microcontroller for the management of sensor data and control over a solenoid valve. This whole system was designed to be a standalone addition to an existing incubator, allowing the user to add it to equipment without making any permanent modifications and subsequently lowering its bar of implementation for initial proof-of-concept research projects.

To allow for the continuous functioning of CyanoStat in a safe and controlled manner, safety features are taken into consideration. These features account for the external implementation of the system, leading to more potential points of user-error induced failure than a fully integrated system. An additional environmental CO_2_ sensor can be added to continuously monitor the concentration within the working space. If a leak develops anywhere between CyanoStat and the incubator, this sensor will sense it and close the flow of gas. Combined, these safety checks add the required reliability for prolonged operation in a high-traffic laboratory.

As a way to adapt the device, adjustable settings have been clearly laid out in the firmware with accompanying explanations. Input of these settings through a user interface (UI) was considered but ruled out to minimize the cost as well as potential points of failure for the finalized product. Additionally, the set-it-and-forget-it mentality that describes the intended use of CyanoStat does not call for frequent setting changes after the initial setup of the system. A UI was thus deemed to be an excessive complexity.

### 2.3 Validation of control strategies for CO_2_ regulation

Three distinct setups of CyanoStat were tested for accuracy and CO_2_ concentration stability over time. These variations of the device implement different response strategies to regulate the required gas flow, namely a threshold control (TC), timed-activation control (TAC), and proportional–integral–derivative (PID) systems. Adaptations between the TC and TAC system were solely done on a firmware level while the PID system required alternate electronics and hardware to build.

**Fig. 1:**
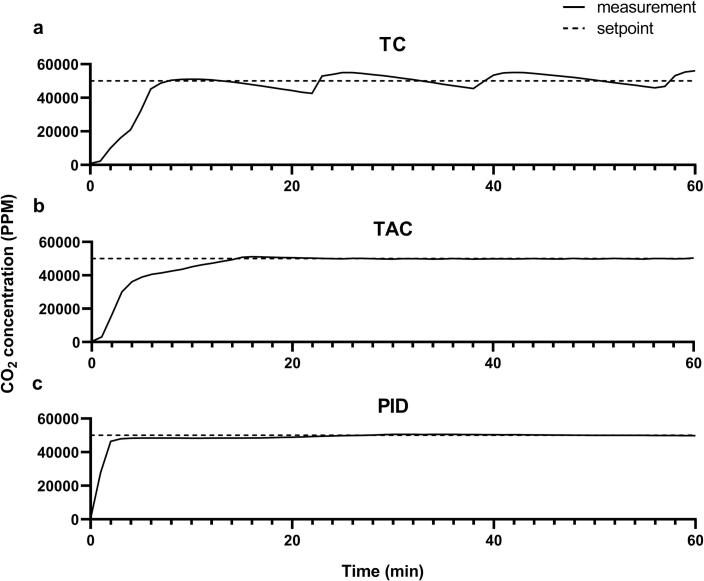
Measurement of CO_2_ concentrations over time using different control strategies. (a) Fluctuations in CO_2_ concentration caused by the bang-bang control strategy. (b) Fluctuations in CO_2_ concentration caused by the time-activated control strategy. (c) Fluctuations in CO_2_ concentration caused by the PID control strategy. PID values used: P = 20, I = 0.6, D = 5. All measurements were started from an off-state (around 450 ppm) and ran for one hour at a setpoint of 50000 ppm. Measurements were taken every 60 s. Graphs are representative of standard system behavior.

The TC system uses the simplest sensor-to-response cascade, taking the concentration measurements and determining the state of the bang-bang controlled valve based on if these values are above or below a threshold value. When CO_2_ concentrations fall below the threshold, consisting of the setpoint minus a pre-determined hysteresis value for stability, the valve will be opened and allow for the influx of gas. When the response is measured to surpass this value, the valve is subsequently closed. Because the response is directly linked to the real-time sensor readings, this control methodology is susceptible to delays between initiating an action and the sensor registering it, leading to significant fluctuations. Initial tests with this system revealed an average fluctuation of approximately 10 % of the setpoint value (Fig. 1**a**).

The TAC system uses a control strategy that incorporates a predetermined response time after the sensor detects a condition, ensuring controlled activation of the response mechanism. Unlike the TC system, which immediately switches a system on or off based on sensor input, the TAC cascade delays the response to mitigate overshoot. The magnitude of the response is adjusted based on the distance of the measured value from the setpoint: when the measured value is far from the setpoint, the valve is opened longer to quickly approach the desired concentration. As the measured value approaches the setpoint, the time diminishes to prevent overshooting. After executing the response, the system waits for the sensor to accurately measure the resulting change before considering any further actions. This approach aims to optimize system stability and performance by balancing the speed of response while minimizing oscillations and maintaining precision around the setpoint. When comparative tests were done in relation to the above-mentioned TC system, a 10-fold reduction in fluctuation around the setpoint was obtained, emphasizing the need for time-based response calculation when implementing low-cost CO_2_ sensors (Fig. 1**b**).

Finally, a PID-controlled system was evaluated to determine whether its increased stability justifies the added cost. While a greater stability is theoretically possible, the system might be hindered by the long time the MH-Z16 sensor takes to register a correcting action, and the required parts increase the cost of the system by more than 4-fold. An SMC PVQ31-6G-23 proportional valve was used, which enables the modulation of the valve's opening proportionally to the current applied to it. When the valve receives 160 mA it will start to open, increasing in a linear fashion towards the maximum flowrate when 330 mA is applied, making it possible to precisely control the CO_2_ flow to an incubator using a PWM signal from the microcontroller. Initial setup of the PID-based system required laborious tuning of the three control variables to obtain reliable control over CO_2_ concentrations, making the setup much less user friendly. After extensive optimization, resulting in PID values of P = 20, I = 0.6, and D = 5, stability measurements were done for an hour (Fig. 1**c**). Since no systems are present to vent excessive CO_2_ from the incubator, these PID settings were calibrated to minimize overshoot past the setpoint. This makes the system initially stabilize at 4.8 % CO_2_ for the first 20 min after being turned on before slowly reaching stability at 5 % after 50 min.

Comparison of the three control schemes clearly shows the disadvantage of the TC control strategy over the others. The prolonged time taken to measure changes after an action is taken makes it so that the fluctuations around the setpoint are comparatively large. Adjusting the calculation method for gas addition in the TAC system allowed us to decrease fluctuations by a factor of ten while retaining the same hardware. The slight increase in initial calibration time required for reliable control using this scheme is thus greatly countered by the increase in performance. The PID control scheme showed greater stability in the long term, eventually barely fluctuating around the setpoint. However, this comes at the expense of responsiveness to big changes in gas concentrations, taking almost a full hour to settle into this stable state. Additionally, the system required extensive tuning to reach this stability. Due to the intricacy of PID tuning, adopting a PID-based system can be difficult for those without background knowledge. Because of this, we decided to use the TAC system for CyanoStat, keeping it affordable and easy to implement while approaching the stability found in a PID-controlled system.

### 2.4 Components

#### 2.4.1 Microcontroller

To control the system, the Seeeduino V4.2 by Seeed Studio was used, a board fully compatible with the Arduino UNO architecture. This board was chosen over the original Arduino UNO because of the multiple improved features it offers at a cheaper price: smaller USB and power connectors for reduced footprint, on-board Grove connectors for I^2^C and UART connections, and switching between 3.3 V and 5 V voltages for the IO. This last feature is essential for this project since the CO_2_ sensors require 3.3 V for their communication with the microcontroller. Due to its compatibility with the Arduino UNO standard, all software and hardware made for this device is transferable to other Arduino UNO compatible boards. To allow an easy readout of measured CO_2_ levels, a 1.3-inch SH1106 I^2^C OLED was connected to one of the Grove connectors. The screen offers a 128x64 resolution, which is sufficient for displaying multiple lines of text or small graphics.

#### 2.4.2 Sensors

The Winsen MH-Z16 nondispersive infrared (NDIR) CO_2_ sensor (hereafter MH-Z16) is used for its low price while offering a range of 0 to 100,000 parts per million (ppm) CO_2_, equating to 0 to 10%. NDIR CO_2_ sensors consist of an infrared (IR) light source combined with a filtered detector. Since IR light is close to the absorption band of CO_2_, increases in concentration can be measured by a relative reduction in the detected radiation. While this specific NDIR sensor is rather large and has a substantial error of 6%, it can be obtained at less than a fourth of the price of more accurate sensors with the same specifications, like the K30 10% CO_2_ sensor, which offers an error rate of 3%. Additionally, it offers UART, PWM, and analog output, a zero-calibration functionality, a response time of T_90_ < 30 s and can be powered by the 5V supplied by the microcontroller.

An optional low-cost Winsen HM-Z19E NDIR CO_2_ sensor (hereafter MH-Z19E) can be added to measure the CO_2_ levels outside the incubator. It’s measuring range of 400 to 5000 ppm (0.04 to 0.5%) is sufficient for monitoring the CO_2_ concentrations in a room, as 5000 ppm is determined to be the Indicative Occupational Exposure Limit (IOEP) by the European union. Due to limitations in the amount of active serial devices handled by the microcontroller, readout of this sensor will happen using a PWM connection.

#### 2.4.3 Solenoid valves

To regulate the flow of gas, an electronically controlled solenoid valve is implemented. A simple and cheap normally closed (NC) valve was used for the TAC-based regulation system. The piston inside this valve fully opens when 12 V is applied to it, allowing gas to flow through it, and closes again when the power is taken away. However, owing to the binary nature of this valve, a system implementing the valve incorrectly struggles to achieve precise concentration control at the desired setpoint. As a result, there would be frequent switching of the valve when partial flow is required to maintain the set concentration, leading to rapid wear on the valve mechanism.

#### 2.4.4 CyanoStat control shield

To make the setup of CyanoStat as simple as possible, a printed circuit board (PCB) was designed to directly slot into Arduino compatible microcontrollers (also known as an Arduino shield). The board offers headers for all peripherals required for CyanoStat, while preserving access to the pin- and Grove connectors. Additional 5 V and 12 V connectors were added for potential future addition of components along with a connector for the optional Z19E environmental CO_2_ sensor. The 12 V power input on the shield PCB supplies power to the valve and is fed into a linear voltage regulator, which provides 5 V to the microcontroller and other peripherals. This setup allows the entire system to operate from a single 12 V power source.

#### 2.4.5 CO_2_ source

Laboratory gas bottles provided by Linde Gas were used for the storage of CO_2_. A pressure regulator was used to adjust the ± 50 bars of pressure down to 0.5 bars before passing through the system. However, access to laboratory gas might be a limiting factor for the usage of CyanoStat. An alternative source of pressurized CO_2_ bottles can be obtained in the form of refills for consumer drink carbonation machines. By utilizing an adapter to convert the T21-4 coupling to a more standard DIN477 connection, after-market pressure regulators can be attached to these bottles. While these bottles only provide up to 425 g of CO_2_, much less than the > 20 kg in industrial-scale bottles, they should suffice for providing a temporary or portable source of gas until other sources are available.

#### 2.4.6 Enclosure

Custom enclosures were designed for the different parts of CyanoStat, allowing for the system to be securely housed in a small box with all the required mounting points and cutouts present. These can be printed with any 3D printer, preferably using the fused deposition modeling (FDM) methodology for its ease and low cost. The models can easily be adapted from the original design files (CAD format) if additional features are added in the future. If a 3D printer is not available, a cheap off-the-shelf project box of at least 100x100x60 mm can be made to fit the main components.

#### 2.4.7 Firmware

**Fig. 2:**
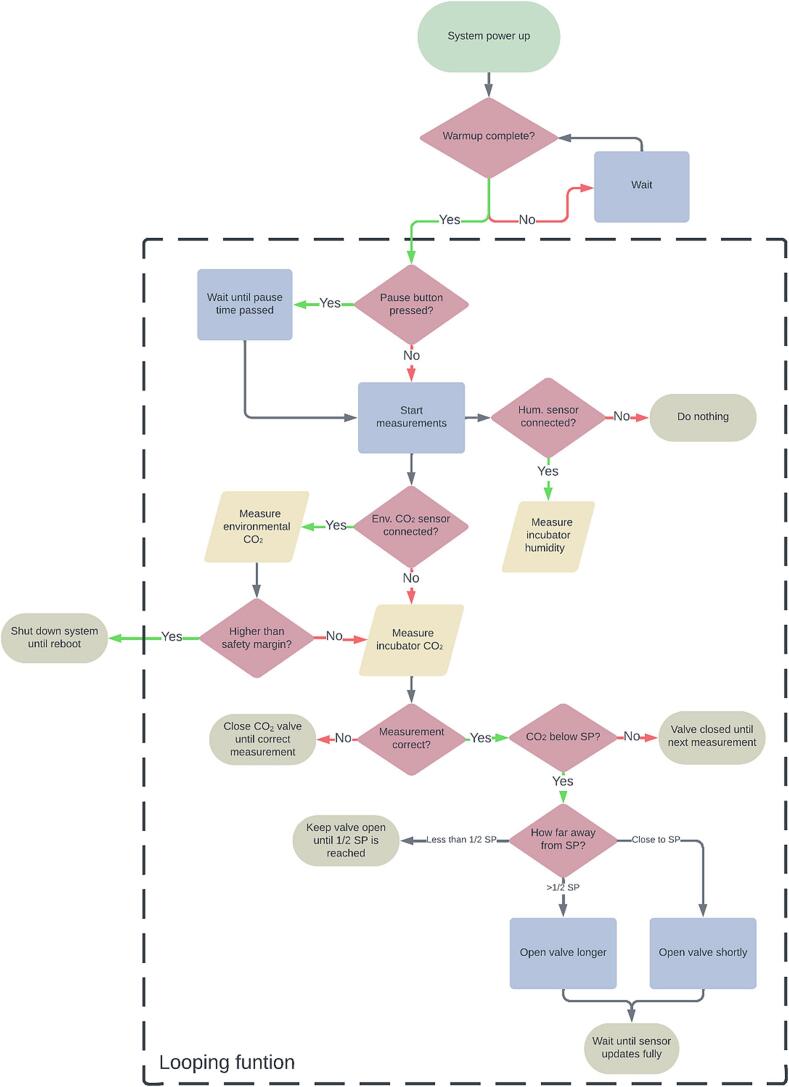
Flowchart of firmware decisions.

To minimize the dependencies needed to compile the firmware, all code required for the reading of CO_2_ values from the MH-Z16 sensor was implemented. This code consists of an adapted and minimized version of the MHZ library by Tobias Schürg. Any variables that can be changed by the user, such as setpoint and presence of optional components, are highlighted near the top of the code. Since the setpoint of CO_2_ within an incubator is rarely changed, the decision was made to only allow these changes to be made over a serial connection, lowering overall cost and complexity of the hardware. The firmware is subdivided in multiple functions used for measurement and safety features (Fig. 2):

- The “Measuring” function reads data from the MH-Z16 sensor and determines whether a response is necessary. If this is the case, it will send a signal to provide 12 V to the solenoid valve, causing it to open and add CO_2_ to the incubator. The duration of the response is determined by the amount the measured value deviates from the desired concentration. Valve opening times can be adjusted using the ShortOpen and LongOpen variables. The function additionally ensures that at least 1020 ms has passed since the last measurement to avoid inaccuracies, as shorter intervals have been found to produce unreliable readings from the MH-Z16 sensor. To account for the 30-second update time needed by the MH-Z16 sensor to accurately detect changes, the function executes a minimum wait time of 60 s after each response before any subsequent corrective action is taken. If the sensor returns the same value for more than a minute, indicating a potential sensor error, the safety system will pause any actions and wait for a change. Additionally, humidity measurements are done by the DHT22 sensor which are displayed on the screen along with the other values.
- The “Buttonpress” function pauses all other functions when activated by the press of the pause button and starts a countdown for the duration given in the setup variables. This allows users to open the incubator without the subsequent drop in CO_2_ concentration causing CyanoStat to take corrective action.
- When the optional MH-Z19E sensor is activated in the setup section, it will execute an additional safety protocol based on environmental CO_2_ readings. If the measured CO_2_ concentration exceeds levels that would typically be expected in a room, suggesting a possible system leak, the valve is closed, a warning is displayed, and the code is paused until corrective action is taken and CyanoStat is rebooted. This function is called once every 30 s from within the “Measuring” function.

## 3 Design files

### 3.1 Design files summary

CAD files for the 3D printed enclosure were created in Onshape 1.182. All CAD files are provided along with STL files for 3D printing (Table 1). 3D models were printed with polylactic acid (PLA) filament using a Bambu Lab A1 3D printer. The development of the PCB was done in EDA KiCad 7.0. Schematics and design files for the CO_2_ control shield are made available for production of PCBs.

**Table 1:** Design- and firmware files required for CyanoStat.

File name	File type	Open-source license	Location of file
CyanoStatBox	STL	CC BY 4.0	https://doi.org/10.17605/OSF.IO/SHD8W
CyanoStatLid	STL	CC BY 4.0	https://doi.org/10.17605/OSF.IO/SHD8W
SensorBox	STL	CC BY 4.0	https://doi.org/10.17605/OSF.IO/SHD8W
SensorLid	STL	CC BY 4.0	https://doi.org/10.17605/OSF.IO/SHD8W
CyanoStatCAD	STEP	CC BY 4.0	https://doi.org/10.17605/OSF.IO/SHD8W
Firmware	INO	CC BY 4.0	https://doi.org/10.17605/OSF.IO/ZDWCG
CyanoStatPCB	KiCAD PCB	CC BY 4.0	https://doi.org/10.17605/OSF.IO/C83FG
CyanoStatSchematic	PDF	CC BY 4.0	https://doi.org/10.17605/OSF.IO/C83FG

## 4 Bill of materials

A bill of materials needed to build a fully functional CyanoStat system can be found in Table 2. While prices are listed for single parts, some components can only be obtained in larger quantities, making the total price slightly higher than the cost of an individual system. The total cost of the parts needed to build CyanoStat is less than €65.

**Table 2:** Bill of materials.

Component	Specification	Unit cost (€)	Total (€)
Microcontroller	Seeeduino v4.2	9.19	9.19
CO_2_ sensor	Winsen MH-Z16 100.000 ppm	32.81	32.81
Humidity sensor	DHT22	1.81	1.81
5-core cable	26AWG, 2 m	1.88	1.88
Power supply	12 V 2A DC, 5.5 × 2.1 mm barrel jack	1.90	1.90
Solenoid valve	W00-05B	9.86	9.86
Tubing	4x6mm silicone tubing, 3 m	1.48	1.48
CO_2_ control shield:			
5 V voltage regulator	L7805CV	0.18	0.18
5 V relay	SRD-05VDC-SL-C	0.36	0.36
50 V Ceramic disc capacitor	1x 0.33uF, 1x 0.1uF	0.01	0.02*
General purpose diode	2x 1 N4007	0.01	0.02
Transistor	BC547 NPN	0.01	0.01
1/4W resistor	1x 1kΩ, 1x 2kΩ, 1x 10kΩ	0.01	0.03
LED	3 mm, red	0.01	0.01
Heatsink	TO220	0.14	0.14
JST-XH socket + connector	4x 2 pin, 1x 4 pin, 2x 5 pin	0.01	0.05*
Single-row female pin socket	2.54 mm pitch, 11 mm length, 1x 6 pin, 2x 8 pin, 1x 10 pin	0.12	0.48*
Barrel jack connector	DCJ200-10	0.14	0.14
PCB manufacturing			0.45*
Enclosure:			
3D printing material	120 g PLA filament	9.60	1.15*
OLED screen	1.3 in., SH1106, 128x64 pixels, I2C	1.68	1.68
Grove to Dupont cable	4 pin, 20 cm	0.60	0.60
JST-XH socket	2x 5 pin	0.01	0.02*
Dupont connector	1x 1 pin male	0.01	0.01*
Push button	6x6x5mm, 4 pin	0.01	0.01*
Mounting hardware	8x M2x10 bolt, 9x M3x8 bolt, 5x M3 nut	0.02	0.44*
Male Dupont connector	1x 1-pin	0.01	0.01
Total:			**64.75**

Optional − Environmental CO_2_:			
CO_2_ sensor	Winsen MH-Z19E 5.000 ppm	13.57	13.57
Single-row female pin socket	1x 4 pin, 1x 5 pin, 2.54 mm pitch	0.09	0.18*

* These components were bought in sets, lowering their individual cost.

## 5 Build instructions

Construction of a CyanoStat system is subdivided into two parts: electronics assembly and firmware adaptation and uploading.

### 5.1 Electronics assembly

#### 5.1.1 PCB assembly

**Fig. 3:**
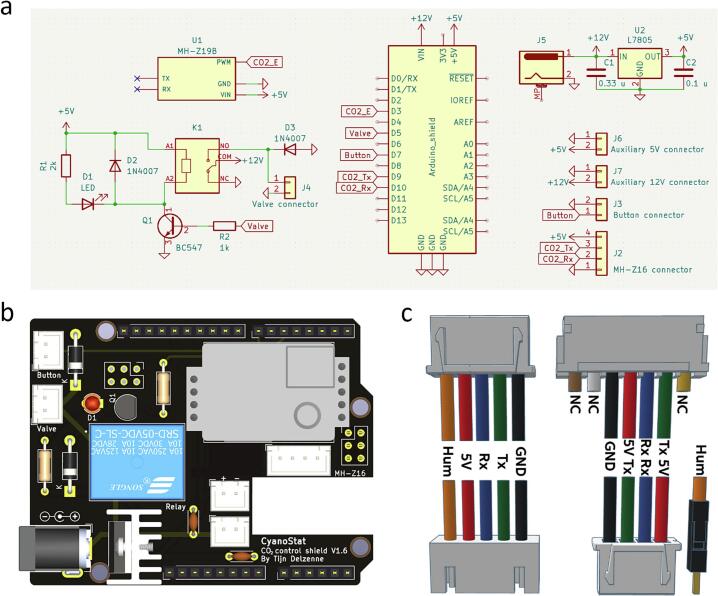
CyanoStat electronics assembly overview. (a) Circuit schematic for the CyanoStat Arduino Uno shield. (b) Overview of the assembled CyanoStat Arduino Uno shield. This version of the PCB has the optional environmental CO_2_ sensor and the headers for auxiliary power (5 and 12 V) mounted. (c) Connector and cable termination overview. Connection coding: Hum = humidity data, 5 V = 5-volt power, Rx = MH-Z16 Rx data, Tx = MH-Z16 Tx data, GND = ground, NC = not connected.

After acquiring all components, they will need to be placed in their designated spots on the PCB. Reference labels in Fig. 3**a** can be compared to markings on the PCB to determine the correct position of each of the components. Start by placing small components on the board, such as resistors and capacitors, and work towards the larger components. For the L7805CV voltage regulator, first attach the heatsink to make sure the spacing is correct when soldering the part to the board. Special attention needs to be paid to the components with polarity (diodes, voltage regulator, and transistor). If the optional environmental CO_2_ sensor is used, it is recommended to mount a 4- and 5-pin single-row connector to the PCB instead of soldering the sensor directly, allowing the sensor to be removed or exchanged in the future if needed. An overview of the finished board can be seen in Fig. 3**b**. For more in-depth information, refer to Supplementary Table S1 and Supplementary Fig. S1**.**

#### 5.1.2 Cable termination

The 5-core sensor cable is stripped on both sides, and the cables are crimped into JST XH (hereafter XH) terminals before being placed in the male connectors (Fig. 3**c**). The 5-core sensor cable is terminated in two male JST XH connectors (Fig. 3**c,** top left) that fit into the female sockets attached to the sensor- and CyanoStat housing (Fig. 3**c,** bottom left). Not every connection on the 7-pin JST PH connector attached to the MH-Z16 sensor is used, hence the zero calibration (brown), V_out_ (white), and PWM (yellow) connections are cut off (Fig. 3**c,** top right). The positive and negative leads of the DHT22 sensor can be soldered together with the respective power leads of the MH-Z16 sensor to allow for the usage of a single cable. The orange data cable is attached to the DHT22 data pin and a 10 kΩ pullup resistor is soldered between the 5 V- and data pin to allow for reliable measurements. For the connections within the CyanoStat housing, five cables are soldered to a female XH connector with the DHT22 data line terminated in a male Dupont connector and the remaining four connections into a four-pin male XH connector (Fig. 3**c,** bottom right). Two cables of around 15 cm are soldered on diagonally opposite contacts of the pushbutton before being crimped and inserted into a 2-pin XH connector. Lastly, the contacts of the solenoid valve are stripped and crimped into another 2-pin XH connector, taking care to match the positive lead to the side that is marked + 12 V on the bottom of the PCB.

#### 5.1.3 Sensor housing assembly

**Fig. 4:**
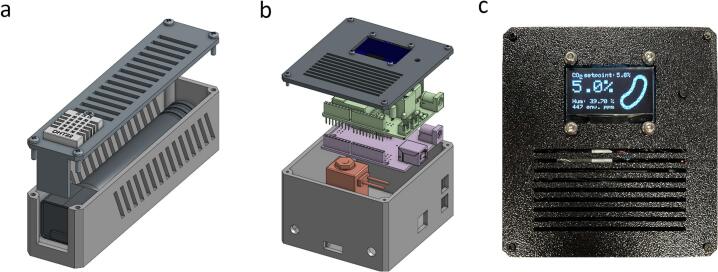
Hardware assembly overview. (a) Exploded view of the sensor housing containing the MH-Z16 CO_2_– and DHT22 humidity sensor. (b) Exploded view of the CyanoStat housing with the main components: microcontroller (pink), electronics shield (green), screen (dark blue), and solenoid valve (red). (c) Photograph of the user interface during operation. Shown from top to bottom: current setpoint, internal CO_2_ measurement, humidity measurement, environmental CO_2_ measurement.

After printing the necessary parts, namely the sensor housing and lid, assembly can start. Two rails present in the housing allow the MH-Z16 sensor to simply and securely slide in without the need for any hardware (Fig. 4**a**). The DHT22 sensor can be secured in place in with an M3x10 bolt and M3 nut. After connecting the cables to the sensors, the lid can be attached to the main housing using four M2x10 screws.

#### 5.1.4 Main housing assembly

After printing the required parts, namely the housing and lid, a dry fitting can be done to see if all parts fit correctly before assembly (Fig. 4**b**). The SH1106 screen should fit snuggly in the designated slot in the lid, whereafter it can be affixed with four M3x8 bolts and nuts. If the tolerances are correctly calibrated on the 3d printer, the push button should be able to be pushed into the lid without the need for any adhesive. Otherwise, a small amount of glue on the side of the switch can be used to hold it in place, taking care to not to get any into the switch itself. The 5-pin female XH connector of the 5-pin to 4-pin + 1-pin internal cable (Fig. 3**c**) is glued into its respective slot in the case, located between the round gas tube ports. Cables are routed internally to avoid obstructing the valve and other electronics. Before screwing the microcontroller into place, ensure that the IO voltage switch is set to 3.3 V. Three M3x8 bolts can be used to screw the microcontroller down. If wiggle room is needed to mount the shield, the screws can be loosened, as the PCB contains holes that allow access for a tool after being installed. All plugs can now be inserted into their respective ports. Connect the screen to the onboard I^2^C Grove connector, checking that the colors of the wires connected to VCC, GND, SDA, and SCL match between the markings on the screen and microcontroller. Connect the gas tubing to the solenoid valve, routing them through the holes in the housing while slotting the valve in place. Make sure to not kink the tubing, which could block the flow of gas. If added, connect the Dupont connector for the humidity sensor to digital pin 6 on the microcontroller and slot the environmental CO_2_ sensor into the pins on the PCB.

### 5.2 Firmware adaptation and uploading

The Arduino IDE is required for adapting and uploading the CyanoStat firmware. In the library manager tab, search for the Adafruit SH110X driver, which is required to be installed to make the SH1106 screen function. If the DHT22 sensor is used, the DHTlib library by Rob Tillaart should additionally be installed before compiling.

At the beginning of the code, a section is designated for setup and calibration variables, allowing the user to have a clear overview of the variables which could be changed (Table 3**,** Fig. S2). While current settings are optimized for the maintenance of 5% CO_2_, the setpoint can be adjusted between 1–6%. When the setpoint is changed, the device automatically adjusts the hysteresis value and valve opening times to compensate (Table 4). The OpenShort and OpenLong variables are calibrated to maintain stable concentrations in an incubator of around 50–60 L. If a larger incubator is used, these values may need to be increased to ensure sufficiently large corrective actions for achieving and maintaining the desired concentration (see section 6.1 for more details). The values of the PWMPin and HumPin definitions should remain unchanged. However, they can be uncommented by removing the two preceding dashes if either the environmental CO_2_ or humidity sensors are utilized.

**Table 3:** Adjustable firmware variables.

Variable	Description	Standard value
Setpoint	Percentage of CO_2_ to be maintained in the system	5
Hysteresis	Percentage fluctuation of the total setpoint allowed	0.5
PauseDuration	Time (s) after pause button is pressed until regulation resumes	60
OpenShort	Time the CO_2_ valve opens every 60 s when big adjustments are needed	6
OpenShort	Time the CO_2_ valve opens every 60 s when small adjustments are needed	2
HumPin	Digital pin the DHT22 sensor is attached to (uncomment if needed)	6
PWMPin	Digital pin the MH-Z19E sensor is attached to (uncomment if needed)	3

**Table 4:** Setpoint calibration settings for a 50-60 L incubator.

CO_2_ setpoint (%)	Hysteresis (%)	OpenLong (s)	OpenShort (s)
1	2	4	1
2	1	4	1
5	0.5	6	2

After connecting an USB cable to the microcontroller, the firmware can be uploaded. A detailed visual guide for this process can be found in Supplementary Fig. S2. If uploading was successful and everything is connected correctly, the startup splash screen should show up on the OLED display. All components except for the valve should work when powered over USB and can be tested while still attached to a computer. After the warmup time, measurements for CO_2_ concentration and, if used, humidity level should be updated every second and an internal LED should light up, indicating that the microcontroller sends a command for the valve to open (Fig. 4**c**). If all the functions are tested to work correctly, the system can be put into action.

## 6 Operation instructions

### 6.1 Usage and calibration

Securely attach the inlet hose of the solenoid to the regulator of the pressurized CO_2_ source. Make sure gas flows into the right port of the valve by referencing the markings on the side. The gas and sensor cables can subsequently be routed through the access port of the incubator, if available. If the incubator has no such port, the cable and hose can be routed via the seal of the door. Testing has shown that minimal gas escapes from an incubator when the cable is routed via the top part of the seal, making it a valid long-term solution. Attach the sensor to the wall of the incubator using the optional 10x2mm magnets in the housing or with adhesive. Open the regulator to a pressure of 0.05 MPa (0.5 bar; 7.2 psi) and check that no leaks are present (see section 6.2).

To activate CyanoStat, connect the 12 V barrel connector to the upper power port. Avoid inserting the connector into the power port of the microcontroller itself, as prolonged use in the incorrect port may damage the system. Allow the system to operate for at least 30 min to verify that the pre-programmed 5% CO_2_ setpoint can be reached and consistently maintained. The firmware automatically adjusts valve opening times for different setpoints based on the required times for maintaining 5% CO_2_, so this setpoint serves as the basis for calibration. The default opening times are optimized for incubators with a volume of 50–60 L, so initial adjustments should be made in proportion to the volume of your incubator. For example, when using a 100–120 L incubator, the opening times should be doubled to accommodate the increased volume. The times should be further increased if the system is not able to maintain the desired setpoint even if a corrective action is taken every 60 s. In this case, large adjustments towards the setpoint will increase the CO_2_ concentration but as soon as the system switches to more precise adjustment, it will be unable to meaningfully change the concentration to reach the setpoint. This behavior can be determined by following the measurement readout on the screen of CyanoStat during initial setup. Opening times should be decreased if large overshoots (> 500 ppm over setpoint) can consistently be perceived after allowing the system to settle in for 30 min. After changing the opening times in firmware and reuploading to the system, allow the device to settle for another 30 min before checking if additional adjustments need to be made.

Once calibration is complete, the setpoint can be modified by connecting the device to a USB port and sending the desired value via serial communication (e.g., to set the CO_2_ level to 2.5%, send “2.5” over serial). The easiest way to achieve this is by using the Arduino IDE, which is also used for uploading the firmware. Open the Arduino IDE and use the Serial Monitor feature to send the command. After the new setting is saved, the updated value, along with the revised hysteresis value and valve opening times, will be confirmed via serial feedback. The system stores setpoints in non-volatile memory, enabling it to retain the settings and resume operation seamlessly after a power cycle.

### 6.2 Safety

An external sensor with alarm should always be used in any room containing equipment using CO_2_ supplementation. This alarm should both be auditory and visual for optimal safety. While a variety of safety features are built into CyanoStat, high concentrations of CO_2_ in a working environment can cause a range of health complications and should just be handled with care [17]. Under operation, any incubator with a CyanoStat system attached should be kept in a well-ventilated area. Before opening the incubator for extended periods, the pause function should be engaged to stop the system from potentially flooding the room with gas. When opening an incubator to access its contents, the user should not lean into the incubator and breathe in the high concentrations of CO_2_ inside. Refer to the safety guidelines for working with CO_2_ set up by your institution or a recognized occupational safety organization, such as OSHA (Occupational Safety and Health Administration), for further safety recommendations and standards.

Notably, some shaking incubators vent gasses out of the incubation chamber, causing CO_2_ to rapidly leak out. Hence, during the installation procedure, environmental CO_2_ levels should be actively monitored with a separate sensor so that such leakage to the surroundings can be ruled out. While the previously mentioned external sensor often suffices for this, a battery-powered handheld CO_2_ sensor can be obtained cheaply for such installation purposes, if needed. Furthermore, we do not recommend installing CyanoStat on a shaking incubator for long periods, as the high humidity caused by incubating liquid cultures combined with elevated CO_2_ levels could form carbonic acid, potentially corroding the motors and electronics over time. The extent of this corrosive effect at different setpoints has not been tested.

## 7 Validation and characterization

### 7.1 Setpoint stability

**Fig. 5:**
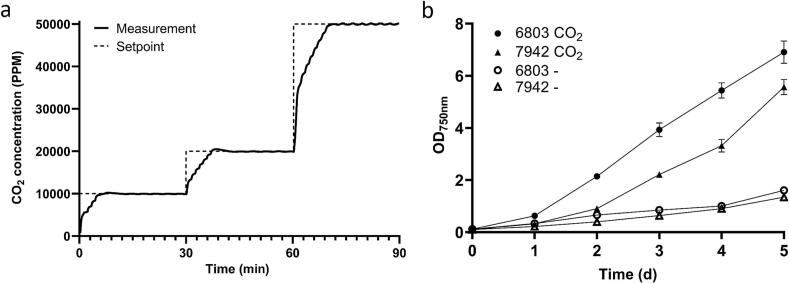
Validation of CyanoStat. (a) Assessment of CO_2_ concentration stability at 10000, 20000, and 50000 ppm. Each setpoint was held for 30 min to allow the system to stabilize. The shown measurements are representative of regular CyanoStat behaviour at these setpoints. The CO_2_ concentration was measured every second. (b) Comparative growth of cyanobacterial species between elevated CO_2_ (2%) and environmental conditions (0.04%). The error bars represent the standard deviation of 3 biological replicates.

While CyanoStat has been optimized for supplementing 5% CO_2_ to an incubator, some users might choose to lower the setpoint to conserve gas or to accommodate non-model cyanobacterial species. Although a CO_2_ concentration of 5% supports optimal growth in lab-optimized cyanobacterial strains [18], this concentration may be excessive for less optimized or environmental samples. To evaluate stability across commonly used setpoints, CyanoStat was operated at 1%, 2%, and 5% CO_2_ for 30 min each, with measurements taken every second (Fig. 5**a**). Hysteresis and valve opening times were automatically adjusted between setpoints in accordance with previous recommendations (see Table 4). At a setpoint of 1% and 2% CO_2_, the maximal deviation after the initial stabilization phase was 1.2% (±120 ppm) and 0.73% (±145 ppm), respectively. Here, fluctuations are expressed as a percentage of the setpoint value, not the total measurement scale. At a setpoint of 5% CO_2_, bigger fluctuations can be seen due to the increase in valve opening times close to the setpoint, mandated by the ShortOpen variable. While these fluctuations could be decreased, this was found to cause frequent valve actuation, reducing the lifespan of the solenoid. Despite the slight reduction in accuracy, the system achieved high stability, with a maximal fluctuation of 0.52% of the setpoint (±260 ppm), on par with commercial incubators. A more detailed setpoint validation for every whole value from 1% to 6% CO_2_ can be found in Supplementary Fig. S3. After the initial stabilization period on startup or setpoint change, this stability is maintained over long periods of time (Fig. S4), with the system running for months on end during development and testing.

### 7.2 Cyanobacterial growth

Finally, validation of the system for the growth of cyanobacterial cultures was tested to compare growth with- and without CO_2_ supplementation using CyanoStat. For this, 6 cultures each of *Synechococcus elongatus* PCC 7942 and *Synechocystis* sp. PCC 6803 were inoculated at an optical density (OD_750nm_) of 0.1. Three cultures were placed on a shaking plate within a Binder KB53 incubator, equipped with a FECiDA CR600 65 W grow light, and supplemented with 2% CO_2_ while the remaining three flasks were grown in an Eppendorf Innova S44i shaker without elevated CO_2_ as a control group. The 2% CO_2_ setpoint was chosen to conserve gas over long periods of time with only a marginal negative effect on growth speed to be expected over a setpoint of 5% [19]. The irradiance at the surface level of the cultures was measured using an UPRtek PG200N spectrometer and LED light output tuned to 70 µM photons m*^−2^ sec*^-1^ of photosynthetically active radiation (PAR). The cultures were subsequently grown at 38 °C with 150 RPM orbital shaking. Growth was assessed over a period of five days through daily OD_750_ measurements (Fig. 5**b**). From day one onward, both cyanobacterial species grown under elevated CO_2_ conditions showed a significantly higher growth rate than the control at all time points (P < 0.005). After 5 days of growth, OD_750nm_ measurements of *S. elongatus* PCC 7942 and *Synechocystis* sp. PCC 6803 were 4.14- and 4.22-times times higher than the control cultures grown in environmental conditions. This shows that CyanoStat can highly increase the efficiency of cyanobacterial growth through the regulation of CO_2_ concentrations in an incubator.

## 8 Conclusion and outlook

This paper describes the design of an open-source CO_2_ regulation device for microbial incubation. By implementing a time-based control system, we achieved performance comparable to that of a PID-controlled system, while significantly reducing both cost and complexity. Additionally, the design incorporates multiple safety features to address potential failure points, making CyanoStat a reliable long-term addition to a laboratory setup.

Although originally designed for cyanobacterial growth, CyanoStat can be applied to a wide range of applications. For example, eukaryotic microalgae share similar growth requirements with cyanobacteria, making them well-suited for cultivation using the system. With further testing, CyanoStat also shows promise for cultivating mammalian cell lines, which require elevated CO_2_ levels similar to those characterized in this research. Lastly, by integrating appropriate sensors, the platform could be extended beyond CO_2_ regulation to control other pressurized gas sources.

## CRediT authorship contribution statement

**Tijn O. Delzenne:** Writing – review & editing, Writing – original draft, Visualization, Validation, Software, Investigation, Funding acquisition, Conceptualization. **Dennis Claessen:** Writing – review & editing, Supervision, Funding acquisition.

## Declaration of competing interest

The authors declare that they have no known competing financial interests or personal relationships that could have appeared to influence the work reported in this paper.
