# Supplementary material for: CyanoStat: An open-source platform for CO2 regulation in microbial incubators optimized for cyanobacterial cultivation
Source: HardwareX. 2025 Apr 12;22:e00649. doi: 10.1016/j.ohx.2025.e00649 (PMC12181779; doi:10.1016/j.ohx.2025.e00649)
Supplement: Supplementary Data 1 [file mmc1.pdf]

# Supplementary data - CyanoStat: An Open-Source Platform for $CO_2$ Regulation in Microbial Incubators Optimized for Cyanobacterial Cultivation

**Supplementary Table S1** - overview of PCB markings and associated parts.

| Marking | Component | Specification |
|---|---|---|
| **C1** | Capacitor | Ceramic disk, 0.33uF |
| **C2** | Capacitor | Ceramic disk, 0.1uF |
| **D1** | LED | 3mm, red |
| **D2,3** | Diode | 1N4007 |
| **R1** | Resistor | 2kΩ, 1/4W, axial |
| **R2** | Resistor | 1kΩ, 1/4W, axial |
| **Q1** | Transistor | BC547 NPN, TO92 |
| **J1,3,4,5** | Connector | JST XH, 2-pin, 2.5mm pitch |
| **J2** | Connector | DC barrel, DCJ200-10 |
| **J6** | Connector | JST XH, 4-pin, 2.5mm pitch |
| **K1** | Relay | SRD-05VDC-SL-C |
| **U1** | Sensor | Winsen MH-Z19E |
| **U2** | Voltage regulator | L7805CV, 5V 1.5A output |
| **P1** | Pin header | 1X10, 2.54mm pitch, 11mm pin length |
| **P2,3** | Pin header | 1X8, 2.54mm pitch, 11mm pin length |
| **P4** | Pin header | 1X6, 2.54mm pitch, 11mm pin length |

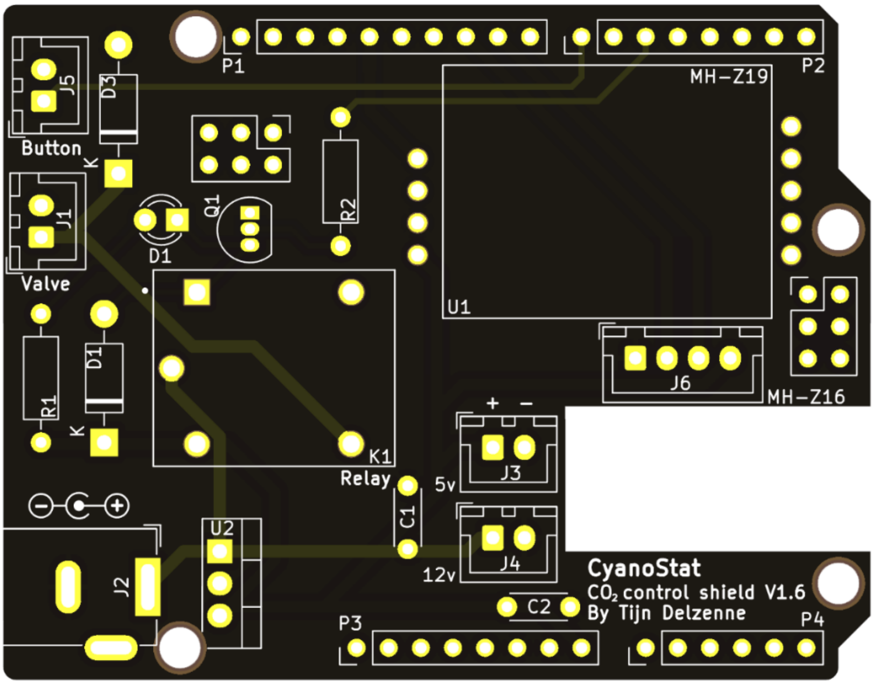


**Supplementary Figure S1** – overview of the unpopulated PCB. Labels on the board correspond to the parts detailed in Supplementary Table 1.

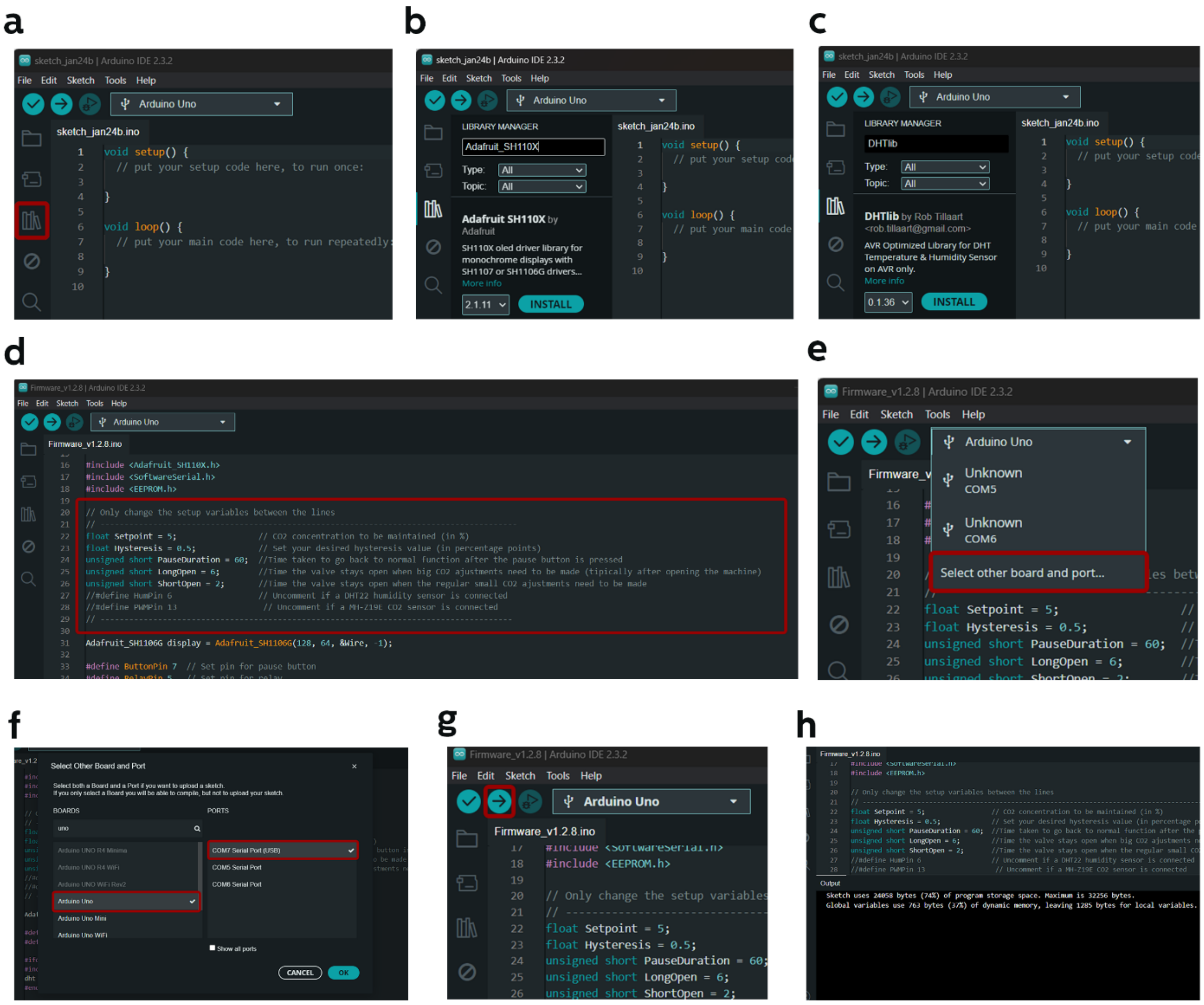


**Supplementary Figure S2** – visual overview of firmware adaptation and uploading. Arduino IDE version 2.3.2 was used.

## Firmware adaptation and installation

**Fig. S2** demonstrates the steps needed to adapt and upload the firmware. After opening the Arduino IDE, two dependencies can be installed from the library tab (a, red box). The Adafruit SH110X library is required for the display to function and version 2.1.11 was used during development (b). The DHTlib library by Rob Tilaart (v0.1.36) is needed to interpret the data from the DHT22 humidity sensor (c). When the dependencies are installed, the Firmware_v1.2.8 file can be opened. All the variables needed to adapt the system are situated at the top of the code with a short explanation of their function (d, red box). When humidity measurements are required and/or the optional environmental $CO_2$ sensor is connected, their functions can be activated by removing the dashes ('//') in front of the definition. After opening the board and serial selection menu (e), the board can be set to the Arduino Uno (f, left red box). Select the right serial connection from the menu to which the firmware should be sent (f, right red box). If you are unsure which one to choose, disconnect the cable to see which COM-port disappears. After the right board and COM-port are selected, upload the firmware (g, red box). If the upload was successful, output should be similar to image h.

**a**

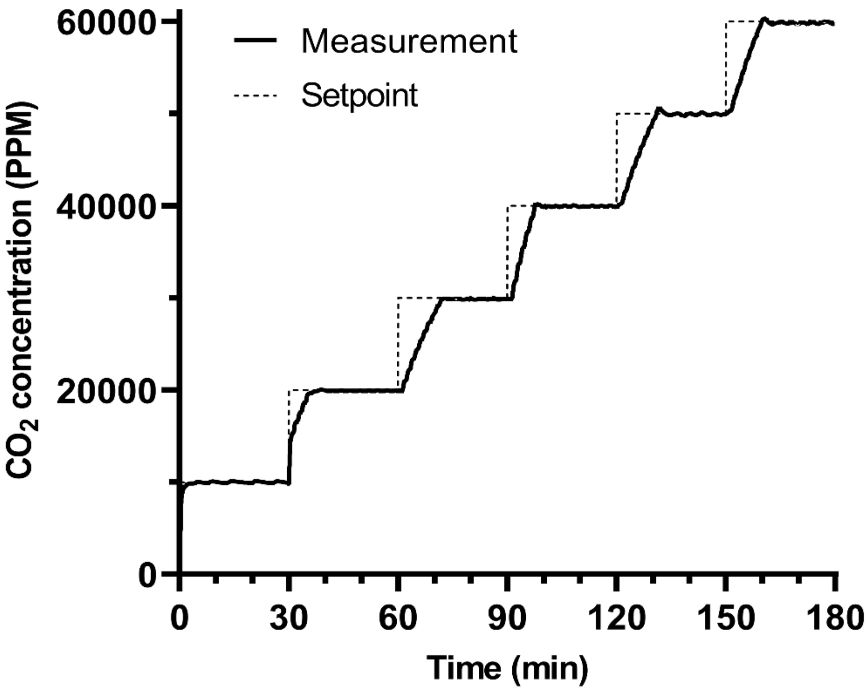


**b**

| SP | Max. fluctuation |
|---|---|
| 1% | ±1.06% (106 ppm) |
| 2% | ±0.56% (111 ppm) |
| 3% | ±0.62% (187 ppm) |
| 4% | ±0.36% (144 ppm) |
| 5% | ±0.34% (171 ppm) |
| 6% | ±0.49% (295 ppm) |

**Supplementary Figure S3** – detailed setpoint validation. (a) Assessment of $CO_2$ concentration stability at setpoints between 1 and 6%. Each setpoint was held for 30 minutes to allow the system to stabilize and subsequently determine the characteristics. The shown measurements are representative of regular CyanoStat behavior at these setpoints. The $CO_2$ concentration was measured every second. (b) Values for the maximal fluctuation from the setpoint (SP) measured in graph **a.** Fluctuation was determined between minute 15 and 30 after a setpoint change to allow for adequate stabilization. Fluctuations are calculated as a percentage of the setpoint value.

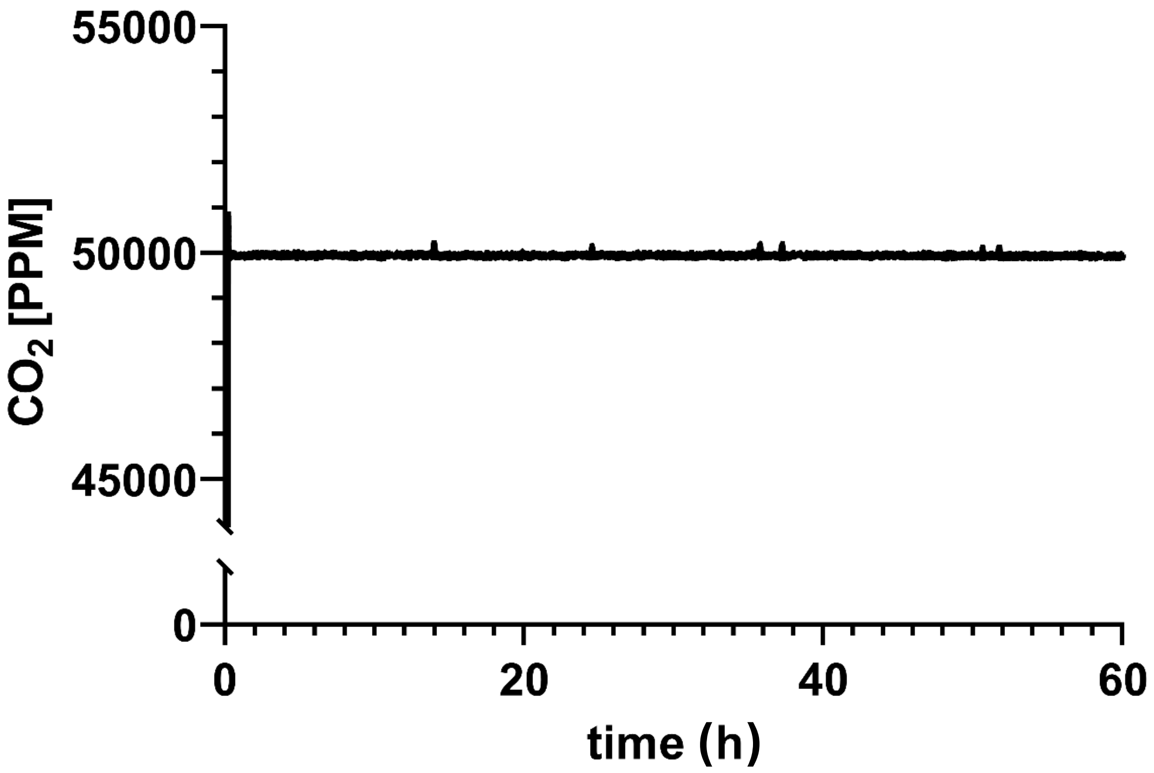


**Supplementary Figure S4** – long-term $CO_2$ stability measurements. $CO_2$ concentrations brought forward by the control of CyanoStat at a setpoint of 5% were measured every second over a period of 60h. Behavior measured after the initial settling time is representative of the stability seen over periods of months during usage. Small overshoot peaks (~500ppm) can sporadically be seen, brought forward by two subsequent corrective actions being taken before the full extent of the initial action is measured. These slight elevations are resolved again within 2 minutes, causing their effect on growth to be minimal.
